# A policy mapping analysis of the U.S. Congressional approach to medical aid-in-dying

**DOI:** 10.1093/geroni/igab046.3263

**Published:** 2021-12-17

**Authors:** Nancy Kusmaul, Ji Hyang Cheon, Allison Gibson

**Affiliations:** 1 University of Maryland, Baltimore County, Baltimore, Maryland, United States; 2 University of Maryland Baltimore, University of Maryland Baltimore, Maryland, United States; 3 University of Kentucky, University of Kentucky, Kentucky, United States

## Abstract

Oregon was the first state to legalize medical aid-in-dying (MAID), in 1994. Since then eight states and Washington, DC have legalized MAID through legislation. Despite literature exploring the legal and ethical aspects of MAID, very little research examines MAID policy at the federal level. This study aimed to 1) examine the objectives of MAID legislation introduced to the US Congress, and 2) investigate whether these bills increase or decrease access to MAID. This study used the congress.gov website to search for bills related to MAID introduced by the US Congress between 1994 and 2020. From the 98 bills identified, we excluded bills that were not directly related to MAID or were introduced in subsequent congresses. In total, 23 bills were retained and analyzed. The greatest number of bills aimed to restrict funds for MAID, followed by bills that sought to regulate the drugs used for MAID. Other bills prohibited the development of policies supporting MAID, regulated penalties for practitioners related to the drugs used for MAID, and restricted legal assistance for accessing MAID. These bills intended to block or limit patient access to MAID by restricting drugs, funds, health care services, legal assistance, policy, and research. These findings suggest that the federal approach is incongruous with the growing numbers of states that have legalized MAID. Federal policymakers must develop policies to 1) prevent discrimination against vulnerable groups, 2) support funds to study MAID, and 3) build a system to allows eligible individuals to access MAID equally.

